# Evaluation of *in vivo* Antiplasmodial Activities of extracts of *Morinda morindiodes* (Bak.) in the treatment of malaria in Ogun State

**DOI:** 10.1186/1475-2875-9-S2-P51

**Published:** 2010-10-20

**Authors:** Soniran O Temidayo, Idowu Olufunmilayo, Idowu A Babatunde, Ajana Olusegun

**Affiliations:** 1 Department of Biological Sciences, University of Agriculture, Abeokuta

## 

*In vivo* study of various plant parts extracts of *Morinda morindiodes* (Bak.) was conducted to evaluate their antiplasmodial properties and effects on the liver using chloroquine sensitive *Plasmodium berghei* in mice. Water extract of the root was observed to significantly reduce parasitaemia (70%, P<0.05) compared to the activities of other plant parts and the untreated control. A mean survival time of 19 days observed in the root extract supported its antiplasmodial activities compared with other plant parts. The antiplasmodial activities of the plant extracts when administered twice daily were not significantly different (P > 0.05) compared with those treated once daily. The chemosuppression produced by the extracts were significantly different compared to untreated control. Liver function tests (LFT) of uninfected mice administered with the plant extracts showed that extracts of the leaf and stem in 'fermented maize starch extract' altered the function of the liver significantly compared to normal mice. This study shows that *Morinda morindiodes* possess antimalarial properties and the root may be used as a prophylaxis where western medicine is not easily accessible and affordable. Tables 1, 2, 3, 4

**Table 1:** Chemosuppression and survival time of *P. berghei* infected mice treated orally with *Morinda morindiodes* extracts at a dose of 100mg/kg body weight once a day for 5 days

Plant part	Extract	%chemosuppression of parasitaemia at day 5 (C.P.±S.D)	%chemosuppression of parasitaemia at day 11 (C.P.±S.D)	Mean Survival time (days)
Leaf	MeOH	7.5 ± 2.8^b^	82.4±5.6^e^	19.5
	Water	10±2.8^bc^	72.6±8.4^bc^	16.5
	F.M. starch	7.5 ± 2.8^b^	78.8 ± 9.9^d^	16.5
Stem	MeOH	0 ± 2.8^a^	60.8 ± 5.6^a^	16.5
	Water	1.3 ± 1.41^ab^	72.2 ± 14.8^bc^	15.5
	F.M. starch	5.0 ± 2.8^b^	71.6 ± 0.0^bc^	16.5
Root	MeOH	20.5 ± 22.6^bc^	67.3 ± 7.07^b^	18.5
	Water	52.5±2.8^d^	76.5 ± 0.0^cd^	19.5
	F.M. starch	30±8.5^c^	79.1 ± 0.0^d^	19.5
Chloroquine		100 ± 0.0^e^		28.5
Artesunate		100 ± 0.0^e^		28.5
Control		0.0		14.5

MeOH, methanol extract; F.M. starch, "aqueous fermented maize starch ('omidun') extract".

**Table 2:** Chemosuppression and survival time of *P. berghei* infected mice treated orally with *Morinda morindiodes* extracts at a dose of 100mg/kg body weight twice a day for 5 days

**Plant part**	**Extract**	**%chemosuppression of parasitaemia at day 5 (C.P.±S.D)**	**%chemosuppression of parasitaemia at day 11 (C.P.±S.D)**	**Mean Survival time (days)**

Leaf	MeOH	20 ±16.9a	83.7±8.5^c^	21.5
	Water	5.0 ± 5.6^ab^	76.1 ± 1.4^bc^	17.5
	F.M. starch	27.5 ± 2 8^abc^	79.4 ± 24.1^bc^	17.5
Stem	MeOH	1.3 ± 11.3^a^	73.2 ± 25.4^b^	16.5
	Water	2.5 ±28.2^a^	71.2±2.8^b^	16.5
	F.M. starch	10.0±5.6^ab^	76.5 ± 9.9^bc^	17.5
Root	MeOH	35 ± 5.6^bc^	80.4±28.2^bc^	19.5
	Water	70.0 ± 2.8^de^	85.9±8.4^bc^	21.5
	F.M. starch	56.2 ± 7.1^cd^	85.6±8.4^c^	17.5
Chloroquine		100 ± 0.0^e^		28.5
Artesunate		100 ± 0.0^e^		28.5
Control		0.0		14.5

MeOH, methanol extract; F.M. starch, "aqueous fermented maize starch ('omidun') extract".

**Table 3:** Comparison between the liver function tests in mice treated with extracts and control group (untreated)

	Leaf extracts		Stem extracts		Root extracts		Contrl	
	
Test	WL	F.M.L	WS	F.M.S	WR	F.M.R	INT.
**Treated Once Daily**							
Total protein(g/l)	53^a^	58.8^b^	61.7^c^	62.4^cd^	63.5^cd^	52.8^a^	65.1d
Cholesterol (mg/dl)	88.7^b^	102.3^d^	106.9^e^	95.2^c^	87.2^b^	63.6^a^	106.9e
SGOT (iu/l)	66^c^	67^c^	85^d^	91^e^	67^c^	30^a^	44b
SGPT (iu/l)	17^ab^	20^b^	18^b^	13^a^	28^c^	25^c^	27c
Urea (mg/dl)	24.5^a^	28.35^c^	25.7^ab^	26.5^bc^	26.5^bc^	28.35^c^	28c
Alkaline phosphatase (iu/l)	95^e^	80^d^	78^c^	47^ab^	40^a^	62b
**Treated twice Daily**							
Total protein(g/l)	60.8^c^	50.4^b^	76.7^e^	46^a^	75.5^e^	65.9^d^	65.1d
Cholesterol (mg/dl)	98.2^f^	75.4^b^	83.6^c^	92.7^e^	60.9^a^	87.1^d^	106.9g
SGOT (iu/l)	43^c^	92^d^	36^b^	93**^d^**	35^b^	19^a^	44c
SGPT (iu/l)	23^c^	22.5^c^	15^ab^	23^c^	12^a^	17^b^	27d
Urea (mg/dl)	22.5^a^	25.05^b^	27.3^c^	25.1^b^	24.2^ab^	24.2^ab^	28C
Alkaline phospatase (iu/l)	29^a^	58^d^	63^d^	37^b^	34^ab^	60^cd^	62d

WL, water extract of leaf; F.M. L, aqueous fermented maize starch extract of leaf.

**Table 4:** Phytochemical analysis of the various plant parts of *Morinda morindiodes*

	Plant Parts / Quantity of Compound		
**Investigated Compounds**	**Leaf**	**Stem**	**Root**

Alkaloid (g/100g)	1.42	1.96	1.62
Saponin (g/100g)	25.3	26.1	22.5
Tannin (mg/100g)	46.2	49.2	38.55
Flavonoid (mg/100g)	14.2	10.4	12.1
Glycocyanides (mg/100g)	**0.98**	1.06	1.12
